# Supplementary material for: Impact of Dosimetric Parameters on Tumor Control in Stereotactic Radiotherapy for Pancreatic Cancer: A Prospective Study on 104 Patients Treated with Simultaneous Integrated Protection (SIP)
Source: Cancers (Basel). 2025 Nov 10;17(22):3617. doi: 10.3390/cancers17223617 (PMC12651346; doi:10.3390/cancers17223617)
Supplement: Supplementary file 1 [file cancers-17-03617-s001.zip › supplementary Table S1.pdf]

Supplementary Table S1. Reasons for unresectability, comorbidity, chemotherapy and detail of Ca19.9 for each patient enrolled in the study

PTS	AGE	ECOG PS	TUMOR STAGE	CA 19.9 AT DIAGNOSIS	UNRESECTABILITY CRITERIA A B OR C	MDT REASONS FOR UNRESECTABILITY	INDUCTION CHEMO	Restaging post cht
1	63	0	CT4CN0	MISSING DATA	A	INVASION OF SMA	mFOLFIRNOX	PR
2	76	1	CT4CN0	56	A+C	FRAIL PATIENTS WITH HIGH COMORBIDITY INDEX + SMA INVASION	0	
3	76	1	CT4CN0	235	A+C	FRAIL PATIENTS WITH HIGH COMORBIDITY INDEX + VEIN INVASION	0	
4	73	1	CT4CN0	543	A	INVASION OF VEIN AXIS NOT AMENABLE FOR RECONSTRUCTION	GEM-ABX	SD
5	73	1	CT4CN0	325	A	VENA CAVA INFILTRATION	GEM-ABX	SD
6	79	1	CT2CN0	218	C	SEVERE HISTORY OF CARDIOPATY WITH REDUCED EJECTION FRACTION	0	
7	79	1	CT2CN0	34	C	END STAGE KIDNEY DISEASE	0	
8	76	2	CT3CN0	87	C	SEVERE PSICHIATRIC COMORBIDITY	0	
9	76	2	CT3CN0	189	C	SEVERE COPD WITH HIGH ANESTESIOLOGIC RISK	0	
10	77	1	CT4CN0	MISSING DATA	A+C	FRAIL PATIENTS WITH HIGH COMORBIDITY INDEX + VEIN AXIS INVASION	0	
11	28	0	CT4CN0	403	A	INVASION OF SMA	mFOLFIRNOX	PR
12	39	0	LOCAL RELAPSE	86	A	NA	mFOLFIRNOX	PR
13	78	1	CT4CN0	74	A+C	FRAIL PATIENTS WITH HIGH COMORBIDITY INDEX + HEPATIC ARTERY INVASION	0	
14	57	1	CT4CN0	346	A	INVASION OF SMA	mFOLFIRNOX	SD
15	57	1	CT4CN0	30	A	INVASION OF SMA	mFOLFIRNOX	SD
16	77	1	LOCAL RELAPSE	MISSING DATA	A	NA	0	
17	77	1	CT4CN0	96	A+C	FRAIL PATIENTS WITH HIGH COMORBIDITY INDEX + SMA	0	
18	69	1	LOCAL RELAPSE	79	A	NA	0	
19	72	1	LOCAL RELAPSE	MISSING DATA	A	NA	0	
20	62	0	LOCAL RELAPSE	32	A	NA	0	
21	62	0	LOCAL RELAPSE	MISSING DATA	A	NA	0	
22	72	2	CT4CN0	MISSING DATA	A+C	FRAIL PATIENTS WITH HIGH COMORBIDITY INDEX + SMA INVASION	0	
23	86	1	CT2CN0	385	C	ELDERLY SUPPOSED NOT TO TOLERATE AT LEAST DOUBLET CHEMO	0	
24	64	1	LOCAL RELAPSE	212	A	NA	0	
25	64	1	LOCAL RELAPSE	MISSING DATA	A	NA	0	
26	64	0	CT4CN0	396	A	INVASION OF VEIN AXIS NOT AMENABLE FOR RECONSTRUCTION	mFOLFIRNOX	SD
27	65	0	CT4CN0	64	A	INVASION OF HEPATIC ARTERY	GEM-ABX	SD
28	43	0	LOCAL RELAPSE	MISSING DATA	A	NA	0	
29	43	0	LOCAL RELAPSE	123	A	NA	0	
30	43	0	LOCAL RELAPSE	MISSING DATA	A	NA	0	
31	45	0	LOCAL RELAPSE	MISSING DATA	A	NA	0	
32	73	0	LOCAL RELAPSE	MISSING DATA	A	NA	GEM-ABX	SD

33	86	1	CT2CN0	65	C	ELDERLY SUPPOSED NOT TO TOLERATE AT LEAST DOUBLET CHEMO	0	
34	84	1	CT4CN0	MISSING DATA	A+C	ARTERY INVASION + AGE	0	
35	84	1	CT4CN0	68	A+C	ARTERY INVASION + AGE	0	
36	62	1	CT4CN0	MISSING DATA	A	INVASION OF SMA	0	
37	67	0	LOCAL RELAPSE	52	A	NA	0	
38	67	0	LOCAL RELAPSE	49	A	NA	0	
39	80	1	CT2CN0	MISSING DATA	C	FRAIL PATIENTS WITH HIGH COMORBIDITY INDEX	0	
40	80	1	CT2CN0	176	C	END STAGE KIDNEY DISEASE	0	
41	52	0	CT4CN0	128	A	INVASION OF VEIN AXIS NOT AMENABLE FOR RECONSTRUCTION	GEM-ABX	PR
42	67	1	CT4CN0	279	A	INVASION OF VEIN AXIS NOT AMENABLE FOR RECONSTRUCTION	GEM-ABX	PR
43	75	1	CT4CN0	MISSING DATA	A	INVASION OF VEIN AXIS NOT AMENABLE FOR RECONSTRUCTION	GEM-ABX	SD
44	84	1	CT2CN0	76	C	ELDERLY SUPPOSED NOT TO TOLERATE AT LEAST DOUBLET CHEMO	0	
45	77	1	CT4CN0	45	A+C	FRAIL PATIENTS WITH HIGH COMORBIDITY INDEX + SMA INVASION	0	
46	65	1	CT4CN0	MISSING DATA	A	VENA CAVA INFILTRATION	mFOLFIRNOX	SD
47	65	1	LOCAL RELAPSE	147	A	NA	GEM-ABX	SD
48	78	2	CT3CN0	320	C	FRAIL PATIENTS WITH HIGH COMORBIDITY INDEX	0	
49	78	2	CT4CN0	1680	A+B+C	INVASION OF SMA +FRAIL PATIENTS WITH HIGH COMORBIDITY INDEX + HIGH CA 19.9	0	
50	65	2	CT3CN0	321	C	ANAMNESIS OF SEVERE VASCULOPATY WITH SMA TROMBOTIC OCCLUSION	mFOLFIRNOX	SD
51	69	1	CT4CN0	MISSING DATA	A	INVASION OF VEIN AXIS NOT AMENABLE FOR RECONSTRUCTION	GEM-ABX	PR
52	77	1	CT4CN0	MISSING DATA	A+C	FRAIL PATIENTS WITH HIGH COMORBIDITY INDEX + SMA INVASION	0	
53	77	1	CT4CN0	244	A+C	FRAIL PATIENTS WITH HIGH COMORBIDITY INDEX + VEIN AXIS INVASION	0	
54	76	1	CT4CN0	MISSING DATA	A+C	FRAIL PATIENTS WITH HIGH COMORBIDITY INDEX + SMA	0	
55	74	2	CT3CN0	245	C	PARKINSON'S DISEASE	0	
56	52	1	CT4CN0	239	A	INVASION OF HEPATIC ARTERY	mFOLFIRNOX	SD
57	74	1	CT4CN0	96	A	INVASION OF SMA	GEM-ABX	SD
58	74	1	CT4CN0	MISSING DATA	A	INVASIO OF CELIAC AXIS	GEM-ABX	SD
59	78	2	CT2CN0	58	C	END STAGE KIDNEY DISEASE	0	
60	78	2	CT2CN0	MISSING DATA	C	FRAIL PATIENTS WITH HIGH COMORBIDITY INDEX	0	
61	78	2	CT4CN0	530	A	INVASION OF SMA+CELIAC ARTERY	0	
62	79	1	CT2CN0	63	C	SEVERE HISTORY OF CARDIOPATY WITH REDUCED EJECTION FRACTION	0	
63	53	0	LOCAL RELAPSE	272	A	NA	GEM-ABX	PR
64	74	1	LOCAL RELAPSE	MISSING DATA	A	NA	0	
65	74	1	LOCAL RELAPSE	MISSING DATA	A	NA	0	
66	76	1	CT4CN0	34	A	VENA CAVA INFILTRATION	GEM-ABX	PR
67	75	1	CT4CN0		A		GEM-ABX	PR

68	44	1	CT4CN0	320	A	INVASION OF VEIN AXIS NOT AMENABLE FOR RECONSTRUCTION	0	
69	58	1	CT4CN0	660	A+B	INVASION OF VEIN AXIS NOT AMENABLE FOR RECONSTRUCTION+HIGH CA 19.9	mFOLFIRNOX	SD
70	85	1	CT4CN0	295	A+C	ARTERY INVASION + AGE	0	
71	85	1	CT4CN0	MISSING DATA	A+C	ARTERY INVASION + AGE	0	
72	58	0	LOCAL RELAPSE	427	A	NA	0	
73	82	1	CT2CN0	21	C	SEVERE HISTORY OF CARDIOPATY WITH REDUCED EJECTION FRACTION	0	
74	61	0	LOCAL RELAPSE	MISSING DATA	A	NA	0	
75	61	0	LOCAL RELAPSE	194	A	NA	0	
76	56	0	LOCAL RELAPSE	68	A	NA	0	
77	59	0	LOCAL RELAPSE	MISSING DATA	A	NA	0	
78	59	0	LOCAL RELAPSE	3	A	NA	0	
79	74	1	CT4CN0	36	A+C	FRAIL PATIENTS WITH HIGH COMORBIDITY INDEX + SMA INVASION	0	
80	49	2	LOCAL RELAPSE	1320	A+B	SMA INVASION, HIGH CA 19.9; PERSISTENT AFTER CHEMO	GEM-ABX	SD
81	55	0	LOCAL RELAPSE	MISSING DATA	A	NA	GEM-ABX	SD
82	57	1	LOCAL RELAPSE	MISSING DATA	A	NA	GEM-ABX	SD
83	81	1	CT4CN0	310	A+C	INVASION OF VEIN AXIS NOT AMENABLE FOR RECONSTRUCTION+AGE	GEM-ABX	SD
84	93	2	CT2CN0	MISSING DATA	C	ELDERLY SUPPOSED NOT TO TOLERATE AT LEAST DOUBLET CHEMO	0	
85	75	2	CT3CN0	MISSING DATA	C	REFUSE OF SURGERY	GEM-ABX	SD
86	85	2	CT2CN0	46	C	ELDERLY SUPPOSED NOT TO TOLERATE AT LEAST DOUBLET CHEMO	0	
87	86	2	CT2CN0	8	C	ELDERLY SUPPOSED NOT TO TOLERATE AT LEAST DOUBLET CHEMO	0	
88	83	2	CT2CN0	195	C	ELDERLY SUPPOSED NOT TO TOLERATE AT LEAST DOUBLET CHEMO	0	
89	83	2	CT2CN0	MISSING DATA	C	DIABETIC PATINETS WITH SEVERE ORGANIC DAMAGE (AMPUTATED)	0	
90	73	1	CT4CN0	294	A+C	LIVER CYRROSIS+INVASION OF CELIAC AXIS	0	
91	73	1	CT4CN0	84	A	INVASIO OF CELIAC AXIS	0	
92	66	2	CT3CN0	47	C	DIABETIC PATINETS WITH SEVERE ORGANIC DAMAGE	0	
93	66	2	CT3CN0	13	C	SEVERE INTERSTITIAL LUNG DISEASE	0	
94	81	1	CT4CN0	245	A+C	INVASION OF VEIN AXIS NOT AMENABLE FOR RECONSTRUCTION+AGE	GEM-ABX	SD
95	81	1	CT4CN0	35	A+C	INVASION OF VEIN AXIS NOT AMENABLE FOR RECONSTRUCTION+AGE	GEM-ABX	SD
96	62	1	CT4CN0	137	A	INVASION OF SMA	GEM-ABX	SD
97	62	1	CT4CN0	421	A	INVASION OF VEIN AXIS NOT AMENABLE FOR RECONSTRUCTION	GEM-ABX	PR
98	82	2	CT2CN0	242	C	ELDERLY SUPPOSED NOT TO TOLERATE AT LEAST DOUBLET CHEMO	0	
99	82	2	CT2CN0	79	C	ELDERLY SUPPOSED NOT TO TOLERATE AT LEAST DOUBLET CHEMO	0	
100	79	2	CT4CN0	5	A+C	FRAIL AND ARTERY INFILTRATION	GEM-ABX	SD

101	79	2	CT4CN0	379	A+C	FRAIL AND ARTERY INFILTRATION	GEM-ABX	SD
102	79	2	CT4CN0	MISSING DATA	A+C	FRAIL AND ARTERY INFILTRATION	GEM-ABX	SD
103	65	2	CT3CN0	169	C	SEVERE HISTORY OF CARDIOPATY AND HYPERTENSION	0	
104	65	2	CT3CN0	95	C	SEVERE COPD WITH HIGH ANESTESIOLOGIC RISK	0	
