# Supplementary material for: Impact of Dosimetric Parameters on Tumor Control in Stereotactic Radiotherapy for Pancreatic Cancer: A Prospective Study on 104 Patients Treated with Simultaneous Integrated Protection (SIP)
Source: Cancers (Basel). 2025 Nov 10;17(22):3617. doi: 10.3390/cancers17223617 (PMC12651346; doi:10.3390/cancers17223617)
Supplement: Supplementary file 1 [file cancers-17-03617-s001.zip › supplementary Figure S1.pdf]

Supplementary Figure S1. Logistic regression showing probability of local control (dependent variable; y axis) in function of PTV_SIP volume (independent variable; x axis). Image shows the trend in decreasing local control probably increasing the PTV_SIP absolute volume.

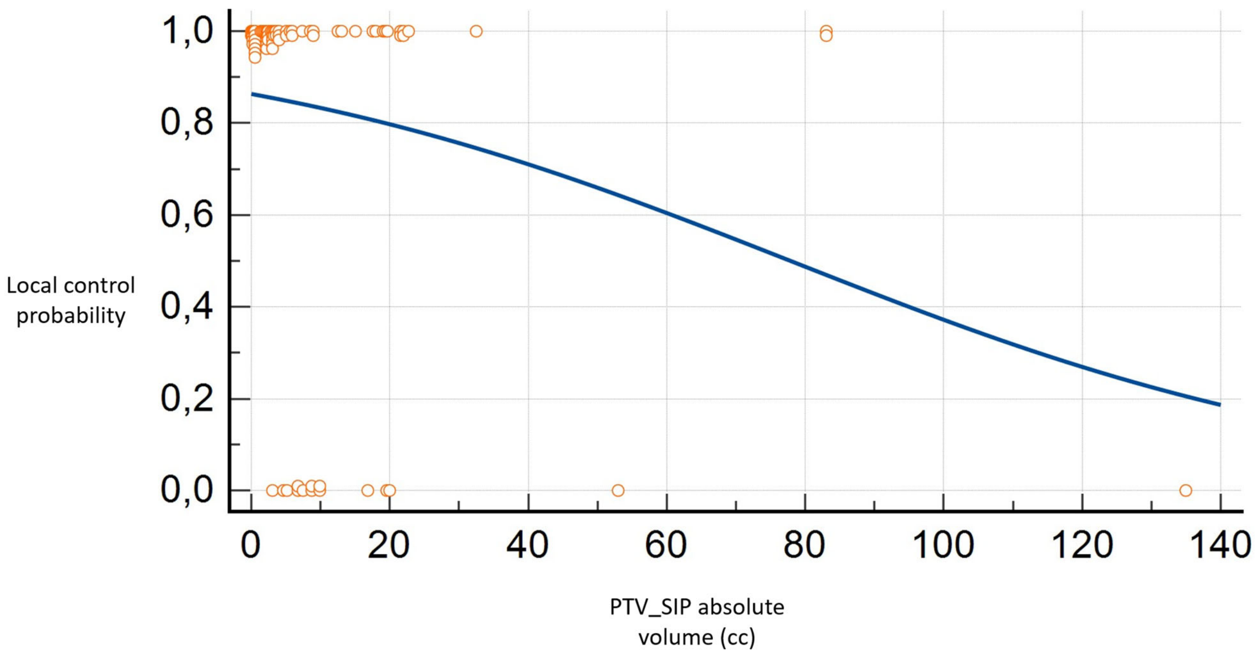

Legenda: SIP=simultaneous integrated protection.
